# Diagnostic accuracy of synovial fluid D-lactate for periprosthetic joint infection: a systematic review and meta-analysis

**DOI:** 10.1186/s13018-021-02778-8

**Published:** 2021-10-16

**Authors:** Zhizhuo Li, Chengxin Li, Guangxue Wang, Lijun Shi, Tengqi Li, Xiaoyu Fan, Xin Xu, Peixu Wang, Fuqiang Gao, Wei Sun

**Affiliations:** 1 Department of Orthopedics, Peking University China-Japan Friendship School of Clinical Medicine, 2 Yinghuadong Road, Chaoyang District, Beijing, 100029 China; 2 Department of Orthopedics, The First Affiliated Hospital of Zhengzhou University, Zhengzhou, 450052 Henan China; 3 Department of Orthopedics, Graduate School of Peking Union Medical College, China-Japan Friendship Institute of Clinical Medicine, 2 Yinghuadong Road, Chaoyang District, Beijing, 100029 China; 4 Beijing Key Laboratory of Immune Inflammatory Disease, China-Japan Friendship Hospital, Peking Union Medical College, 2 Yinghuadong Road, Chaoyang District, Beijing, 100029 China

**Keywords:** D-lactate, Synovial fluid, Periprosthetic joint infection, Arthroplasty, Meta-analysis

## Abstract

**Background:**

Periprosthetic joint infection is a grievous complication after arthroplasty that greatly affects the quality of life of patients. Rapid establishment of infection diagnosis is essential, but great challenges still exist.

**Methods:**

We conducted research in the PubMed, Embase, and Cochrane databases to evaluate the diagnostic accuracy of D-lactate for PJI. Data extraction and quality assessment were completed independently by two reviewers. The pooled sensitivity, specificity, likelihood ratios, diagnostic odds ratio (DOR), summarized receiver operating characteristic curve (sROC), and area under the sROC curve (AUC) were constructed using the bivariate meta-analysis framework.

**Results:**

Five eligible studies were included in the quantitative analysis. The pooled sensitivity and specificity of D-lactate for the diagnosis of PJI were 0.82 (95% CI 0.70–0.89) and 0.76 (95% CI 0.69–0.82), respectively. The value of the pooled diagnostic odds ratio (DOR) of D-lactate for PJI was 14.18 (95% CI 6.17–32.58), and the area under the curve (AUC) was 0.84 (95% CI 0.80–0.87).

**Conclusions:**

According to the results of our meta-analysis, D-lactate is a valuable synovial fluid marker for recognizing PJI, with high sensitivity and specificity.

## Introduction

Arthroplasty is an effective treatment for end-stage joint diseases. With the continuous improvement of the surgery, complications are gradually reduced but are still unavoidable; of these, periprosthetic joint infection (PJI) is the most serious complication [1].

More than 2% of patients undergoing arthroplasty experience PJI, which is the culprit in the failure of total knee arthroplasty and the third most common indication for hip revision [2]. At the same time, PJI has brought heavy lifestyle and economic burdens to patients and the health care system. After the diagnosis of PJI, two treatment options are available, debridement-and-retention and revision, which cost 3 and 3.4 times more than the cost of primary implantation, respectively, while the cost of two-stage revision is 1.7 times more than that of one-stage revision [3]. The clinical symptoms of PJI are varied and are affected by many factors, such as the pathogenic organism, host immune response, time of onset, and site of infection. The Infectious Diseases Society of America (IDSA) suggests that one should be alert for the development of PJI after arthroplasty in the presence of the following symptoms: persistent sinus or wound oozing, acute postoperative initiation pain, and chronic postoperative pain.

The most common clinical methods used to diagnose PJI are peripheral blood tests, imaging examination, and microbiology. Conventionally, a definitive diagnosis of PJI requires a combination of clinical symptoms and history, and it cannot draw the conclusion of infection through a single examination. However, due to the influence of many factors, PJI usually has no specific clinical manifestations in the early stage [1]. Organizations such as the Musculoskeletal Infection Society (MSIS), the American Academy of Orthopaedic Surgeons (AAOS), and the European Bone and Joint Infection Society (EBJIS) have proposed several criteria for the diagnosis of PJI [4–6], but the complexity of the definitions limits its application in daily clinical practice. Therefore, a more specific and sensitive routine test to diagnose PJI is urgently needed.

Lactate can be divided into D-lactate and L-lactate, and two lactates are isomers. L-lactate, as a final product of glucose metabolism, is the main form of lactate in the human body, existing in the blood and muscle [7]. D-lactate is produced almost entirely by bacteria in the human body, such as *Escherichia coli*, *Klebsiella pneumoniae*, *Staphylococcus aureus* and fungi, which are specific metabolites of pathogens and can be detected in body fluids and used in the diagnosis of infection [8]. Due to the lack of D-lactate dehydrogenase in tissue cells, the metabolism of D-lactate in the human body is slow, and the level of D-lactate in synovial fluid can be detected by enzyme spectrophotometry [9].

In recent years, increasing attention has been given to the value of synovial fluid biomarkers in the diagnosis of periprosthetic joint infection. Synovial fluid D-lactate was proposed as a novel diagnostic biomarker for PJI by Yermak et al. in [10], and it has also been reported to have high sensitivity and specificity. However, the clinical value of D-lactate in assessing PJI is still under debate and investigation. In recent years, several studies have been published on the application of D-lactate for the detection of PJI. However, because of the heterogeneity of study quality, their results are inconclusive. Therefore, the aim of this systematic review and meta-analysis was to synthesize published data on the accuracy of D-lactate in the detection of PJI and assess the diagnostic value of D-lactate for PJI.

## Material and methods

The research methods and statistical methods used in this article are consistent with the Cochrane Collaboration's diagnostic test accuracy methodology [11]. We report the results of the current systematic review in accordance with the Preferred Reporting Items for Systematic Reviews and Meta-Analyses (PRISMA) criteria [12]. No ethical approval or informed consent was required for this article, as all data were obtained from the published literature. The research method, identification of eligibility, data extraction, and quality assessment were performed independently by two researchers. Any disagreements were resolved through discussion, and consensus had to be reached between the two researchers.

### Search strategy

We searched PubMed, Embase, and the Cochrane Library on June 21, 2021, and adjusted the vocabulary and grammar according to the results, but there was no time limit. We used "periprosthetic joint infection" or "prosthesis-related infection" as the diagnosis of interest and "D-lactate" as our target index. There were no applicable language restrictions. The list of references to related articles was also manually filtered to look for any additional records.

### Inclusion criteria

The following criteria needed to be met for the studies included in this systematic review: (1) population consisting of patients who had undergone arthroplasty, (2) D-lactate test performed in synovial fluid, (3) diagnosis of PJI confirmed by the MSIS, AAOS or EBJIS guidelines and (4) sufficient data that could be extracted for constructing a 2 × 2 contingency table. Case reports, reviews, expert opinions, narrative comments and studies in animals were excluded. If different studies provided overlapping data, only the most comprehensive or up-to-date studies were included.

### Data extraction

The data extracted and recorded in the standardized Excel file included the first author's last name, study inclusion interval, year of publication, country, demographic information of the participants, study design, location of the arthroplasty, number of infected/total joints, method of evaluating D-lactate, cut-off value, diagnostic criteria, and number of false/true positive and false/true negative cases.

### Quality assessment

According to the QUADAS (Quality Assessment of Diagnostic Accuracy Studies)-2 tools for evaluating the methodological quality of the included studies, the analysis includes four key areas (i.e., patient selection, index test, reference standard, and flow and timing). The risk of bias was assessed in each area, and the first three areas assessed the concerns about applicability with signaling questions. The answers to these questions were expressed as "yes," "no," and "unclear." “Yes” indicates a low risk of bias/concern; "No" indicates a high risk of bias/concern; and "unclear" indicates no relevant information was explicitly provided [13].

### Statistical analyses

We calculated the pooled sensitivity, specificity, positive likelihood ratio (PLR), negative likelihood ratio (NLR), and diagnostic odds ratio (DOR) of the extracted data using the bivariate meta-analysis framework. The bivariate model adopts the method of random effects, and the statistical characteristics of the bivariate model are suitable for diagnostic meta-analysis. In addition, the accuracy of the tests was described by constructing pooled receiver operating characteristic (sROC) curves as well as the area under the curve (AUC). The *I*^2^ statistic was used to assess the heterogeneity among the included studies. A value of 0% implies that no heterogeneity was observed, while a value greater than 50% indicates a high degree of heterogeneity. We also performed Deek's funnel plot asymmetry test to determine the presence of publication bias. In all statistical tests, bilateral *P* < 0.05 values were considered statistically significantly different. Stata version 14 (StataCorp, College Station, TX) was used to analyze the included extracted data, and Review Manager software version 5.3 (Cochrane Collaboration, Oxford, United Kingdom) was used to assess the methodological quality of the included studies.

## Results

### Search results and study selection

**Fig. 1:**
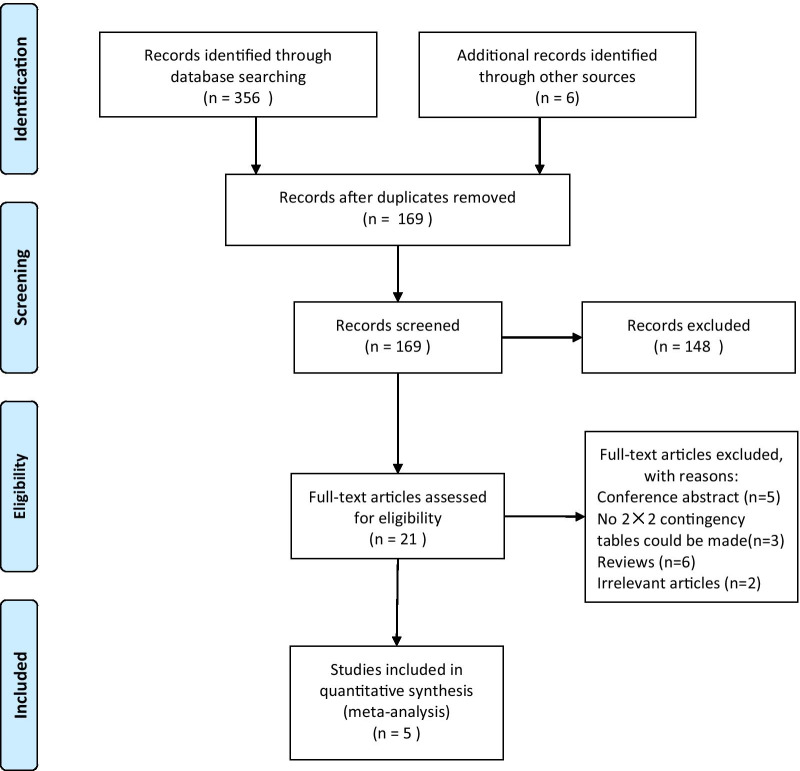
Selection process of included studies

First, we searched the databases and removed duplicate records, identifying a total of 169 records. Then, an initial screening of titles and abstracts was performed, resulting in 21 articles that met the precriteria, and 5 articles [9, 10, 14–16] were finally included for quantitative analysis after further evaluation. Figure 1 shows the selection process of the included studies.

### Study characteristics

Five studies including a total of 1087 patients (253 patients by PJI) were included in the systematic review to explore the diagnostic accuracy of D-lactate; four studies [9, 10, 14, 16] were prospective, and one study [15] was retrospective. Three studies [9, 10, 14] used a D-lactate kit (VL-Diagnostics, Leipzig, Germany) to assess D-lactate, and two studies [15, 16] did not give a test method. The average age of the patients included in the study was 66.4–69.5 years, and the proportion of males ranged from 44.6 to 54.7%. The main characteristics of the included studies are summarized in Table 1.

**Table 1:** Characteristics of the included studies

Study (Published year)	Inclusion interval	Country	Infected/total joints	Male/female	Mean Age (y) (range)	Study design/type	Site of Arthroplasty	Cut-off value	Method of testing	Diagnosis criteria
Karbysheva et al. 2020	2016.03–2018.06	Germany	71/224	100/124	66.4(30–96)	P Cohort	Knee:125 Hip:99	1.3 mmol/L	D-lactam Kit^a^	MSIS
Sharma et al. 2020	2000–2018	USA	50/107	57/50	65.9	P Cohort	Knee:93 Hip:14	NR	D-lactam Kit	MSIS
Yermak et al. 2019	2016.05–2017.03	Germany	44/148	81/67	69.5(29–93)	P Cohort	Knee:103 Hip:43 Shoulder:2	1.3 mmol/L	D-lactam Kit	EBJIS
Lenski et al. 2015	2007.01–2015.12	Germany	67/562	NR	NR	R Cohort	NA	5.3 mmol/L	NR	MSIS
Lenski et al. 2014	NR	Germany	21/46	NR	NR	P Cohort	Knee:47 Hip:19 Elbow:2	8.3 mmol/L	NR	MSIS

P = prospective; R = retrospective; NR = not reported; MSIS = Musculoskeletal Infection Society; EBJIS = European Bone and Joint Infection Society

^a^D-lactam Kit; VL-Diagnostics, Leipzig, Germany

### Results of the quality assessment

**Fig. 2:**
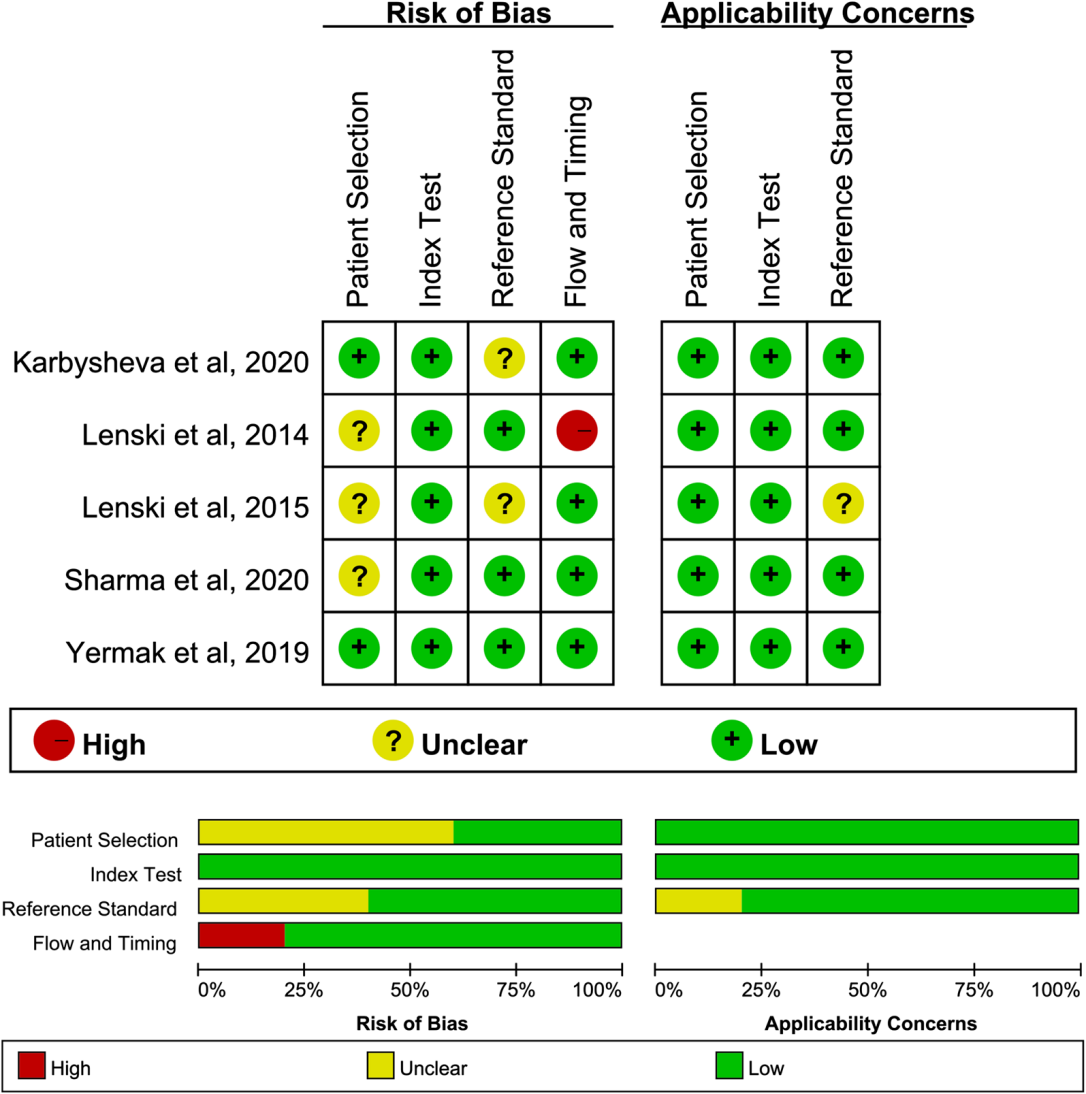
Quality assessment of included studies using QUADAS-2 tool criteria. Red in figure indicates high risk, yellow represents unclear risk, and green means low risk

Figure 2 shows the results of each of the included studies assessed by the QUADAS-2 tool. The quality of the studies included was good, as the percentage of high risk was less than 25% in each key area.

### Diagnostic value of D-lactate for PJI

**Fig. 3:**
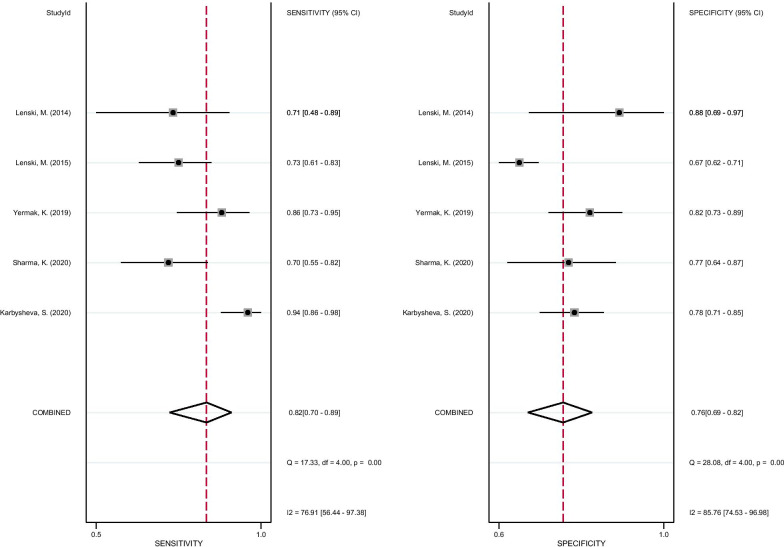
Forest plots of the sensitivity and specificity of D-lactate for periprosthetic joint infection across all included studies

**Fig. 4:**
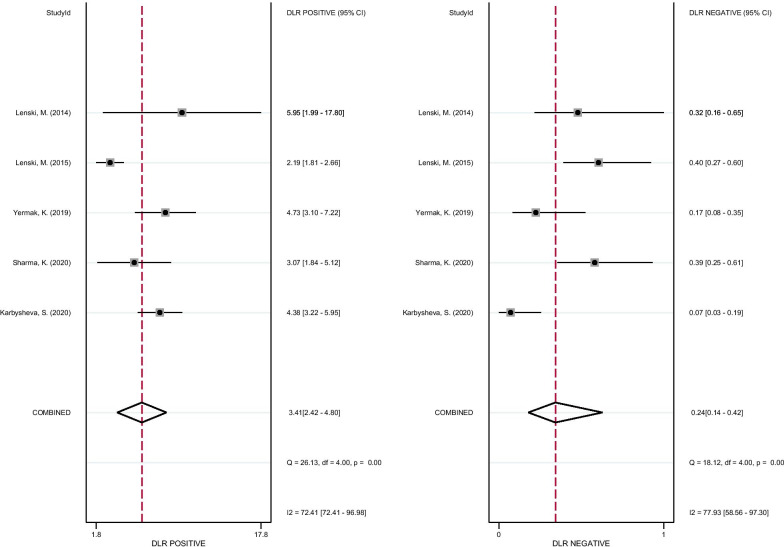
Forest plots of the PLR and NLR of D-lactate for periprosthetic joint infection across all included studies

**Fig. 5:**
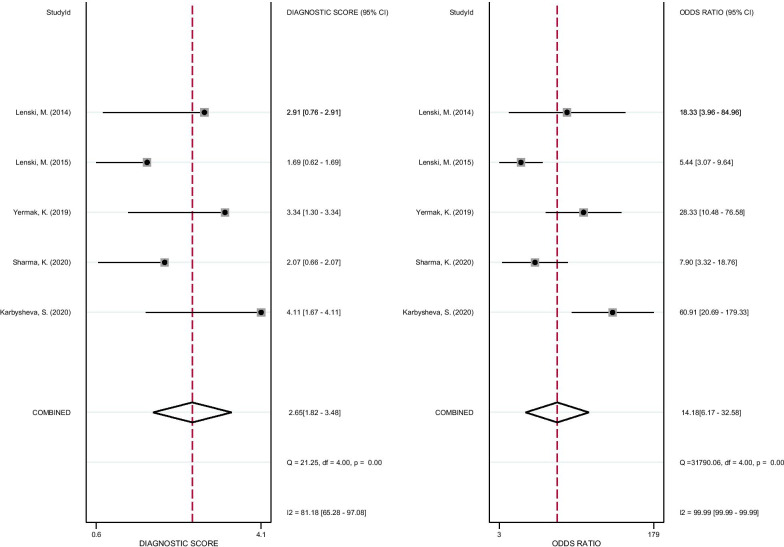
Forest plots of the diagnostic score and DOR of D-lactate for periprosthetic joint infection across all included studies

**Fig. 6:**
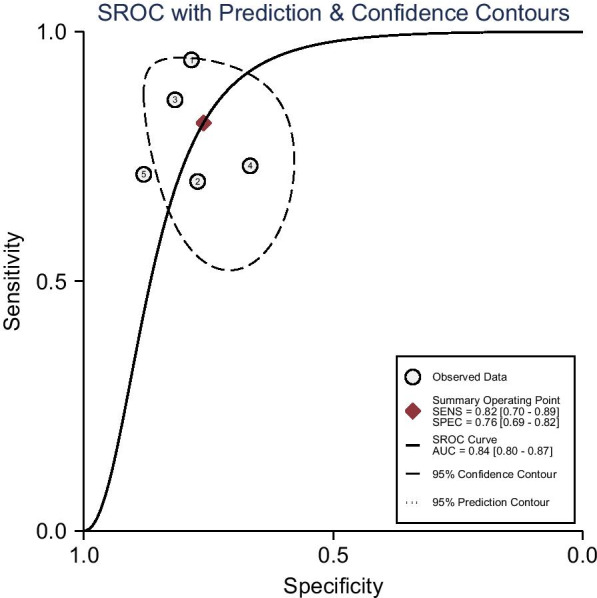
Summarized receiver operating characteristic curve (sROC) of D-lactate for periprosthetic joint infection with corresponding 95% confidence region and the 95% prediction region

**Fig. 7:**
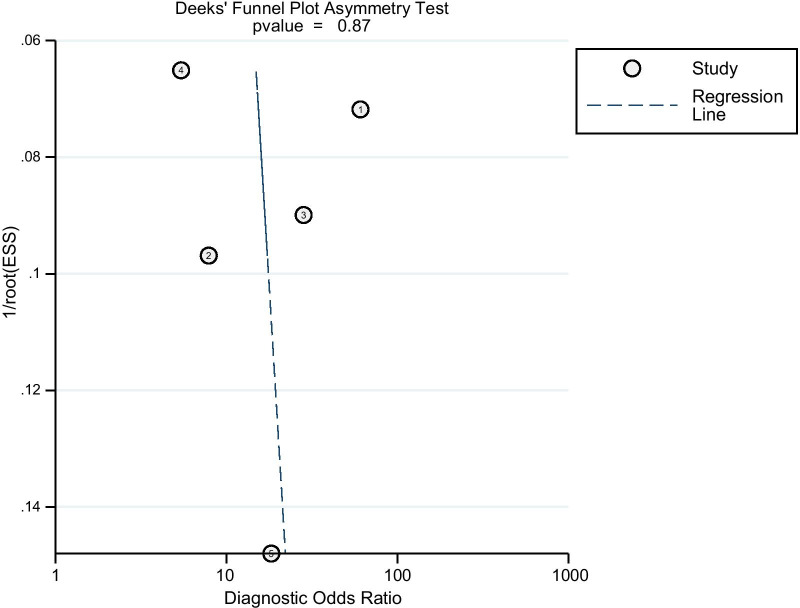
Deek’s funnel plot asymmetry test indicated no evidence of significant publication bias

As shown in Fig. 3, the pooled sensitivity and specificity of D-lactate for the diagnosis of PJI were 0.82 (95% CI 0.70–0.89) and 0.76 (95% CI 0.69–0.82), respectively. The pooled PLR, NLR, and DOR were 3.41 (95% CI 2.42–4.80), 0.24 (95% CI 0.14–0.42), and 14.18 (95% CI 6.17–32.58), respectively (Figs. 4, 5). The AUC for D-lactate against PJI was 0.84 (95% CI 0.80–0.87) (Fig. 6). The I^2^ statistics for sensitivity and specificity values were 76.91% (95% CI 56.44–97.38%) and 85.76% (95% CI 74.53–96.98%), respectively, indicating substantial heterogeneity in the included studies. Pooled data of D-lactate on PJI calculated by STATA and estimates of Spearman's correlation coefficient (*P* value > 0.05) suggest that the heterogeneity was not due to a threshold effect. In addition, we performed Deek’s funnel plot asymmetry test. The result of the test was 0.87, indicating the absence of publication bias (Fig. 7).

## Discussion

At present, there is an increasing number of arthroplasties with an ageing population and medical progress. Periprosthetic joint infection (PJI) occurs in 0.7% of patients [17] and is considered a serious complication associated with considerable morbidity and mortality. Symptoms of aseptic prosthesis failure partially resemble PJI; however, the treatments differ greatly [18]. Therefore, an accurate diagnosis of infection is crucial for appropriate therapy. The Musculoskeletal Infection Society (MSIS) definition criteria for PJI were from 2013 and are widely accepted as the “gold standard” [4]. These criteria included either two major criteria or four of the following six criteria. However, MSIS criteria may miss several low-grade and delayed infections because of the high threshold [19]. To sensitively, specifically and quickly diagnose septic arthritis, several synovial fluid host-specific biomarkers, including interleukin-6, adenosine deaminase, alpha-defensin, leukocyte esterase, and calprotectin, were evaluated. As these biomarkers are all abundantly present in neutrophils, aseptic conditions associated with high synovial fluid leukocyte count, such as crystal-induced arthritis, posttraumatic inflammation, or rheumatic joint diseases, can also see the increase [20, 21].

D-lactate, a pathogen-specific metabolite, is produced nearly exclusively by bacteria [22]. It has been previously evaluated in primarily sterile body fluids, including cerebrospinal and synovial fluid. D-lactate and L-lactate are isomers. However, D-lactate in the human body is slow due to the lack of D-lactate dehydrogenase. In contrast, D-lactate is produced almost entirely by bacteria, which can be detected in body fluids and used for specific diagnosis of infection. Moreover, delayed infections are known to evoke only subtle clinical symptoms and signs of low microbial burden. As bacterial metabolism decreases with biofilm maturation, detectable amounts of D-lactate are still produced. In addition, the D-lactate concentration seems to depend on the number of bacteria, as the concentration of D-lactate was higher in culture-positive PJI than in culture-negative PJI. Therefore, different detection concentrations can reflect various clinical implications [10].

Our study revealed that D-lactate was highly sensitive and specific in identifying PJI by applying MSIS criteria (pooled sensitivity and specificity of 0.82 and 0.76, respectively), indicating a comparable, extremely high diagnostic ability to identify PJI using this biomarker. Karbysheva et al. reported that D-lactate is comparable to the synovial fluid leukocyte count [9]. Advantages of the D-lactate test are the low volume of synovial fluid required (50 μL), quick turnaround time (45 min), and low expense (calculated on actual production costs). The high sensitivity and rapid availability of results make this biomarker particularly useful as a point-of-care screening test for PJI. Karbysheva et al. also diagnosed PJI and evaluated treatment success with D-lactate according to modified Zimmerli criteria [23]. They found that the optimal D-lactate cut-off was 1.2 mmol/L (sensitivity = 98%, specificity = 84%). D-lactate has better sensitivity for the diagnosis of PJI (98%) than leukocytes and neutrophils (80% and 89%, respectively, *P* < 0.0001). The concentration of D-lactate decreased below the cut off within four weeks after revision surgery, showing relapse of infection (*P* < 0.0001). D-lactate has the best sensitivity as an independent diagnostic method and could be implemented for the evaluation of treatment success.

The strengths of the current study lie in the following two aspects. First, this is the first meta-analysis of D-lactate for the diagnosis of PJI. The results show that this method has the advantages of accuracy, economy and rapidity. It provides a new reference index for clinical practice and has important clinical significance. Second, our results point out the direction for future research, which indicates that the D-lactate concentration is likely to reflect the virulence and microbial load of pathogenic bacteria. At present, the relationship between the concentration of D-lactate in synovial fluid and the virulence of bacteria is not clear and needs further study.

Potential limitations of this meta-analysis should also be considered. First, our study only compared the results of D-lactate with MSIS guidelines. Second, the cut-off values were different, and there was no subgroup analysis, so the results were not uniform. Third, the included articles lacked prospective RCTs, resulting in a low quality of the results.

## Conclusions

Based on the results of this meta-analysis, it could be concluded that joint fluid D-lactate has significant potential value for the diagnosis of periprosthetic joint infections with high sensitivity and specificity. Moreover, the test is very convenient and can be performed preoperatively or intraoperatively.

## Data Availability

The datasets used and/or analyzed during the present study are available from the corresponding author on reasonable request.
